# Velocity-Based Strength Training: The Validity and Personal Monitoring of Barbell Velocity with the Apple Watch

**DOI:** 10.3390/sports11070125

**Published:** 2023-06-23

**Authors:** Basil Achermann, Katja Oberhofer, Stephen J. Ferguson, Silvio R. Lorenzetti

**Affiliations:** 1 Section Performance Sport, Swiss Federal Institute of Sport Magglingen (SFISM), 2532 Magglingen, Switzerland; basil.achermann@baspo.admin.ch (B.A.);; 2 Institute for Biomechanics, ETH Zurich, 8092 Zurich, Switzerland

**Keywords:** resistance training, velocity-based training, barbell kinematics, sensor technology, inertial measurement unit (IMU), wearables

## Abstract

Velocity-based training (VBT) is a method to monitor resistance training based on measured kinematics. Often, measurement devices are too expensive for non-professional use. The purpose of this study was to determine the accuracy and precision of the Apple Watch 7 and the Enode Pro device for measuring mean, peak, and propulsive velocity during the free-weighted back squat (in comparison to Vicon as the criterion). Velocity parameters from Vicon optical motion capture and the Apple Watch were derived by processing the motion data in an automated Python workflow. For the mean velocity, the barbell-mounted Apple Watch (r = 0.971–0.979, SEE = 0.049), wrist-worn Apple Watch (r = 0.952–0.965, SEE = 0.064) and barbell-mounted Enode Pro (r = 0.959–0.971, SEE = 0.059) showed an equal level of validity. The barbell-mounted Apple Watch (Vpeak: r = 0.952–0.965, SEE = 0.092; Vprop: r = 0.973–0.981, SEE = 0.05) was found to be the most valid for assessing propulsive and peak lifting velocity. The present results on the validity of the Apple Watch are very promising, and may pave the way for the inclusion of VBT applications in mainstream consumer wearables.

## 1. Introduction

Resistance training (RT), which involves working against an external load to increase muscle mass, maximal strength, and power output, has been shown to offer a range of benefits for both athletes and recreational exercisers [1,2,3,4]. Various methods for monitoring training intensity have been introduced, including those based on subjective questionnaires and user ratings, as well as objective measures based on the heaviest moveable weight, i.e., one repetition maximum (1RM), or velocity data, also named velocity-based training (VBT) [5,6]. While linear position transducers (LPT) are reliable [7] and considered the gold standard for measuring barbell velocity, they are often too expensive for non-professional use [8,9,10]. Recent studies have therefore investigated the validity and reliability of more affordable technologies, such as cameras, smartphone apps, and wearables designed specifically for VBT [11,12,13]. However, a specific area of interest that has not been sufficiently explored is the potential use of consumer wearables capable of measuring barbell velocity for VBT, while also providing additional features (i.e., sleep tracking). Despite the growing popularity of these devices among athletes and recreational exercisers, their effectiveness and accuracy in this specific context remain largely unverified [14].

Wearable devices equipped with inertial measurement unit (IMU) sensors have gained popularity in the sports sciences community due to their convenience and accessibility [15]. Harnessing the potential of a smartwatch for VBT measurements could provide a practical and affordable method for athletes and recreational exercisers to monitor their training intensity and performance. The integration of VBT with health and fitness applications could further enable the development of personalized training regimes, better tracking of fitness progress, and enhanced fitness outcomes. 

However, IMUs are known to exhibit random errors [16]. Thus, the validity of commercially available IMUs for VBT monitoring is still discussed in the field. Multiple studies have assessed the validity of different IMU-based VBT devices such as the Beast Sensor (Beast Technologies S.r.l., Brescia, Italy), VmaxPro (BM Sports Technology GmbH, Magdeburg, Germany) and Push Band 2 (Whoop, Boston, MA, USA) [11,12,17,18,19] in a variety of free weight and Smith machine-supported barbell exercises. No definitive conclusion has yet been drawn, thus highlighting the need for further investigation. It remains to be validated whether consumer wearables such as smartwatches, equipped with IMU sensors and exemplified by the Apple Watch (Apple Inc., Cupertino, CA, USA) [20,21], have the capacity to accurately measure barbell kinematics during free-weighted back squats.

To address this research gap, the primary goal of the present study was to validate the Apple Watch as a tool for measuring barbell kinematics in VBT against data from optical motion capture as gold standard. We assessed its performance during a VBT protocol on healthy, recreational RT athletes by comparing the resulting velocity data of the free-weighted back squat from two different watch positions (i.e., wrist and barbell), compared with Vicon (Vicon 3D Motion Systems, Oxford, UK) as a criterion [22]. As a secondary objective and for a comprehensive and practically oriented assessment, we further compared the results from the Apple Watch with a commercially available IMU-based VBT device, namely the Enode Pro device (Blaumann and Meyer Sports Technology UG, Magdeburg, Germany), formerly known as Vmaxpro [12,19,23,24]. Our aim was to provide scientifically robust, praxis-oriented insights into the validity of the Apple Watch for monitoring barbell kinematics during VBT, given its widespread popularity as a consumer electronic device and the growing interest in VBT among athletes and recreational exercisers.

## 2. Materials and Methods

The study protocol complied with the Declaration of Helsinki for Human Experimentation and was approved by the regional ethics committee (Kantonale Ethikkommission Bern, Nr: 2021-00403). The study design was developed to assess the validity of the Apple Watch 7′s IMU sensor during free-weighted back squats in healthy volunteers. The methodology employed in this study was similar to previous validation studies [8,11]. All participants gave written signed consent prior to any data acquisition.

### 2.1. Participants

The participants in the present study were 22 recreationally active RT athletes with at least 3 years of experience in performing free-weighted back squats (n = 12M/10F, age = 29.1 ± 5.2, height = 1.65 ± 0.4 m; body mass = 77.5 ± 12.6 kg; back squat 1RM = 134 ± 32 kg). Written informed consent was obtained from each participant prior to data acquisition. All participants were healthy and regularly physically active. 

### 2.2. Study Design

**Figure 1:**
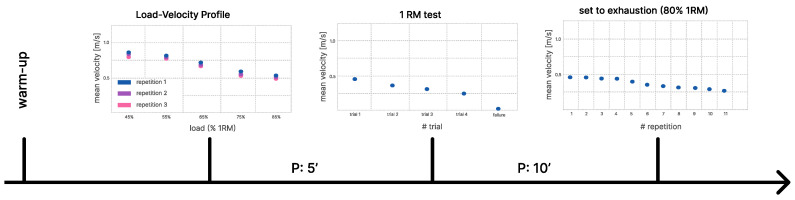
Visualization of testing protocol. It comprised a warm-up, the assessment of each individual’s load–velocity profile, an actual 1RM test, and a set performed to exhaustion.

Subjects reported to the lab for one testing session, where they performed back squats between 45 to 100 percent of their one-repetition maximum (1RM) at maximum voluntary lifting velocity, according to VBT guidelines [25]. The testing protocol comprised a warm-up, the assessment of each individual’s load–velocity profile, an actual 1RM test, and a set performed to exhaustion. Details of the testing protocol are shown in Figure 1 and further outlined in the following paragraph.

Each participant performed a 10 min individual warm-up according to their standard training routine, which comprised cycling or rowing, followed by dynamic stretching and warm-up exercises using an empty barbell (20 kg), such as good mornings or back squats. Following the warm-up, a load–velocity profile was firstly assessed for each participant, by instructing them to squat three times with maximum lifting velocity with a loading of 45, 55, 65, 75 and 85% of their estimated 1RM. The participants were instructed to pause for 3 min between each load. Secondly, after a pause of 5 min, the actual 1RM of each participant was assessed. Here, the choice of load and pause between attempts was left to the participants to improve their self-confidence before the lift. In case of failure, a second attempt was given, depending on the feedback and fatigue level of the participant. Thirdly, after a pause of 10 min to ensure the best possible recovery, a set-to-exhaustion with 80% of the achieved 1 RM was performed. Participants were explicitly guided to pause momentarily between repetitions, ensuring that a brief but distinct interval was present between repetitions. This measure was implemented to enhance the separability of individual repetitions for more accurate data analysis.

### 2.3. Data Collection

**Figure 2:**
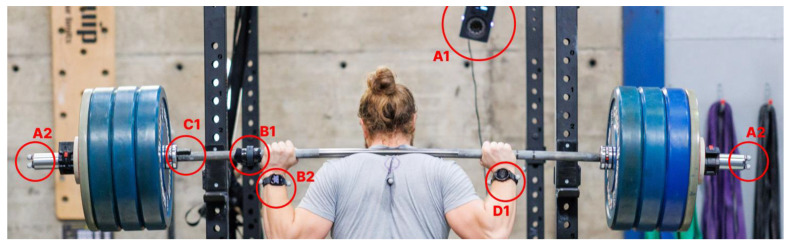
Setup of measurement devices. A1: Vicon infrared camera with A2: reflective markers on both proximities of barbell. B1: barbell-mounted Apple Watch, B2: wrist-worn Apple Watch, C1: barbell-mounted Enode Pro, D1: smartwatch not included in study.

Velocity parameters were independently but simultaneously measured with an optical 3D motion capture system (as the criterion), two Apple Watch 7s (version: 9.0), both wrist-worn and barbell-mounted, and an Enode Pro device (version: 1.1.6, firmware: 5.2.0) (Figure 2).

The barbell-mounted Apple Watch was positioned adjacent to the left hand of the subject, approximately 60 cm from the left extremity of the barbell. Depending on the participants preferences, the position was adjusted slightly. The wrist-worn Apple Watch was firmly affixed to the left wrist to eliminate any device movement during data acquisition.

The Enode Pro was fixed to the outermost place of the inner part of the barbell. For optical motion capture, two reflective markers were attached on both sides of the barbell, still enabling the change of weight plates between tests, with ten infrared cameras (Vantage 5, Vicon Motion Systems Ltd., Oxford, UK) placed around the participant to record the motion of the markers.

The Enode Pro device was operated via a tablet (iPad Pro, Apple Inc., Cupertino, CA, USA) and calibrated according to instructions of the corresponding app. Following calibration, the device remained attached to the barbell and switched on throughout the entire testing session of each subject. For standardization, it was rebooted and recalibrated after each participant, or in the event of any errors.

To facilitate data collection from the Apple Watch, a Node.js server application equipped with an user interface was developed to stream and receive mobile data via the Sensorlog [26] application (version 5.2). The Node.js application was utilized for all interactions with the Apple Watches during the sessions.

To facilitate communication between the Apple Watch and the paired iPhone (iPhone 13 Pro, Apple Inc., Cupertino, CA, USA), a local network was established, and both iPhones were connected to it during the testing session. Thus, the Apple Watch could communicate with the paired iPhone as its parent device through the Node.js server. To manually calibrate the Apple Watch on the wrist prior to each set, participants were instructed to perform a functional calibration movement that involved swinging the arms at self-selected speed for approximately 30 s. This was not utilized any further in this work.

All Vicon cameras were controlled from an Antec WorkBoy desktop (Antec, Taipei, Taiwan) running Vicon Nexus software (version 2.9, Vicon Motion Systems Ltd., Oxford, UK).

### 2.4. Data Processing

Velocity parameters from the Vicon optical motion capture and the Apple Watch were derived by processing the motion data using the procedures outlined in the Supplementary Materials (Criterion: Supplementary Material S1, Apple Watch: Supplementary Material S2). Thereby, both markers on either side of the barbell were used to derive the displacement and velocity at 60 cm from the left side of the barbell. The resulting velocity magnitudes from the optical motion capture were used as the validation criterion for comparison with the velocity data from the Apple Watch. To ensure accurate identification of the concentric phases, all repetitions from both modalities were graphically reviewed. Velocity parameters from the Enode Pro were obtained through the export feature of the corresponding iOS application. In our study, the Enode Pro device encountered some recognition errors, in which repetitions were recorded inaccurately, leading to instances of duplicates or neglection. In case of such errors, the entire set was excluded for statistical analysis. Additionally, a validity check was conducted on the velocity parameters, where the criterion Vpeak (peak concentric velocity) > Vmean (mean concentric velocity) was evaluated prior to statistical analysis.

### 2.5. Statistical Analyses

The validity of the Apple Watch was assessed against the criteria for Vmean, Vpeak and Vprop [27] (propulsive concentric velocity) separately, using the three-tier approach recommended by Hopkins [28]; this approach comprises a Pearson’s correlation coefficient (r), a calibration equation, and the standard error of the estimate (SEE). This analysis was carried out for (1) the whole velocity spectrum, (2) repetitions with a concentric criterion duration below 1.25 s, and (3) those with a concentric criterion duration above and equal to 1.25 s, which were categorized as “total”, “slow” and “fast”, respectively. The time threshold was chosen accordingly, to build two similarly sized groups with two velocity spectra (“slow” and “fast”).

For the calibration equation, ordinary least product (OLP) regression was used [29], as both the criterion and Apple Watch measurements were subject to random measurement errors. To assess the effect of the Apple Watch’s placement, the OLP between the wrist-worn and barbell-mounted Apple Watch was used. The SEE was calculated from residuals of the OLP calibration equation, according to Siegel [30] and Fritschi et al. [12]. Correlation coefficients were interpreted as large, very large, and extremely large for thresholds of 0.5, 0.7, and 0.9, respectively [31].

According to Fritschi et al. [4], SEE values less than 0.1 m/s and greater than 0.3 m/s are considered low and high-precision values, respectively, whereas SEE values between 0.1 and 0.3 m/s are considered moderately precise. The chosen values of 0.1m/s were adequate for identifying a 30% velocity loss at relatively heavy loads. Proportional measurement bias was considered to exist if the 95% confidence limits of the calibration slope did not include 1, while a fixed measurement bias was considered to exist if the 95% confidence limits of the calibration intercept did not include 0 [29]. This issue is discussed further in the subsequent sections, as the use of an OLP with sufficient high precision allows for the correction of measurement biases. All statistical analysis was performed with Python, employing the SciPy [32] and Pingouin [33] libraries.

## 3. Results

### 3.1. Data Set Description

Data from a total of 599 repetitions of the free-weighted back squat in 22 participants were acquired (complete data set in Supplementary S3). After post-processing and elimination of repetitions that exhibited clear errors, the data for validation purposes included 578 (97%), 547 (91%), and 496 (83%) paired observations for the barbell-mounted Apple Watch–Vicon, the wrist-worn Apple Watch–Vicon, and the barbell-mounted Enode Pro–Vicon, respectively (Table 1). The connection between the SensorLog App and the Node.js Server application experienced some timeouts. It was found that this issue was more prevalent for the wrist-worn compared to the barbell-mounted Apple Watch (49 vs. 19 reps). Additionally, the results indicated that the Enode Pro exhibited a higher number of recognition errors compared to the Apple Watch (17 vs. 2–3 reps). Furthermore, 17 repetitions of the Enode Pro failed the validity check, because the Vmean value was larger than the Vpeak velocity.

### 3.2. Validity

#### 3.2.1. Precision

**Figure 3:**
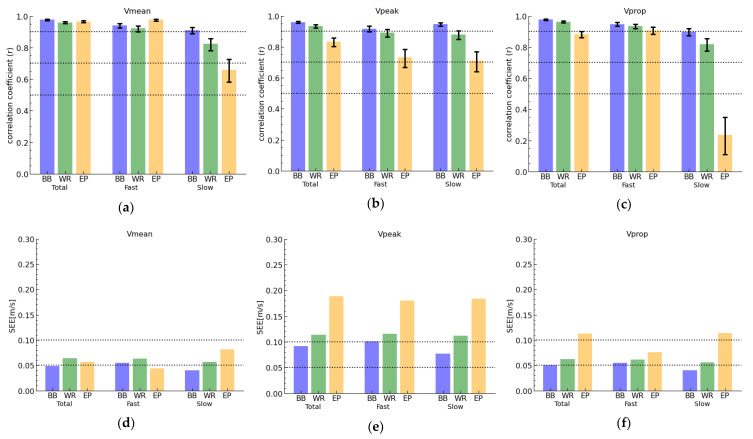
Bar plots indicate the values of the Correlation coefficient (r, top row: **a**–**c**) and standard error of estimate (SEE, bottom row: **d**–**f**) of mean, peak, and propulsive velocity measures (i.e., Vmean, Vpeak, Vprop) for all three devices, i.e., the Apple Watch on the barbell (BB), Apple Watch on the wrist (WR) and Enode Pro (EP), in comparison to Vicon as criterion. As described in the methods, the captured repetitions were categorized as total, fast and slow velocity spectra to be separately analyzed. The 95% confidence interval is visualized via the error bars. The dotted line visualize thresholds which were discussed in Section 2.5 Statistical Analyses.

The indicators of the precision (SEE, correlation coefficient (r)) of all devices are visualized in Figure 3 and in Table 2 (complementary information can be found in Supplementary Material: Tables S1–S3). Additionally, Figure 3 shows the influence of the velocity spectrum on the precision.

The barbell-mounted Apple Watch yielded the highest correlation coefficients (r = 0.879–0.981) in comparison to the Vicon data as criterion, followed by the Apple Watch worn on the wrist (r = 0.774–0.969) and lastly the Enode Pro (r = 0.110–0.981). Particularly for slow velocities, a weak correlation between the Enode Pro and Vicon was found (r = 0.110–0.768). This trend was also confirmed by the SEE values (Figure 3d–f). Here, the values for the barbell-mounted Apple Watch never exceeded 0.1 m/s, closely followed by the wrist-worn Apple Watch, which always had a slightly higher SEE value (0.56–0.114 m/s). The SEE values for Enode Pro were in a similar range for Vmean (0.036–0.078 m/s) and Vprop (0.076–0.114 m/s), but considerably higher for Vpeak (0.180–0.188 m/s).

#### 3.2.2. Accuracy

The parameters for the OLP regression are presented in Table 2. OLP results for the different velocity zones can be found in the Supplementary Materials. The slope coefficients demonstrated a consistent pattern for all repetitions across the full velocity spectra and parameters for both Apple Watches (barbell-mounted and wrist-worn). For both devices, the Vpeak showed a larger divergence to the slope value 1 compared to Vmean and Vprop.

Otherwise, the barbell-mounted Enode Pro device displayed shallower slope gradients for Vpeak and Vprop. The different results in accuracy for the Apple Watches compared to Enode Pro are addressed in detail in the discussion section.

### 3.3. Precision of Wrist Placement

The effects of the placement of the Apple Watch on the precision are presented in Table 3. Precision indicators displayed a high correlation (r = 0.942–0.969) and low SEE values (SEE = 0.06–0.09 m/s) between the wrist-worn and barbell-mounted Apple Watch.

## 4. Discussion

This study examined the validity of two inertial sensors (Apple Watch and Enode Pro) to derive velocity parameters during VBT with free-weighted back squats in healthy, recreational strength training athletes (compared to Vicon as the criterion). To our knowledge, our study is the first to use the IMU sensors of a commercially available smartwatch in a VBT validation study. Furthermore, only a few studies to date have assessed the accuracy of the Enode Pro device across different loads and exercises (including the back squat) compared to the Apple Watch and Vicon as the criterion [12,19,23].

### 4.1. Importance of Precision

It is generally agreed among practitioners that precision is of greater significance than accuracy when monitoring VBT using a specific device [12]. Here, it is important to note that the present segmentation algorithm developed for the Apple Watch did not incorporate any correction of fixed or proportional biases, and that the present findings are specific to the free-weighted back squat exercise during VBT; this is because different exercises and execution guidelines may yield varying levels of precision. The author suggests that potential developers of VBT applications should consider incorporating bias correction into their algorithms to improve inter-device reliability. However, although some slight over- or underestimations may exist, the precision parameters presented in Figure 3 are considered a good measure for the validity rating in this study.

Some studies have reported a decrease in the precision of IMU devices depending on the velocity of repetitions, particularly in overestimations at higher velocities [16,19]. In our work, we observed a loss in precision using the Enode Pro device compared to Vicon, with an underestimation at lower velocities (Figure 3). This loss in precision at lower velocities was not observed using either Apple Watch. The lower precision of the Enode Pro for slow repetitions could be attributed to the fixation mechanism of the device or to the low velocities assessed in this study. The Enode Pro fixation is solely reliant on a magnetic component as opposed to being tightly strapped to the barbell, as is the case with the barbell-mounted Apple Watch and for other Enode Pro studies [12,19,23,34]. The influence of low velocities on IMU precision might be valuable to assess in further studies.

Unlike the slow repetitions, the mean lifting velocity of the Enode Pro achieved higher precision for fast repetitions compared to the Apple Watch, which supports the quality of the IMU sensor. This finding raises the suspicion that the observed errors for slow propulsive (Vprop) repetitions using the Enode Pro might originate from segmentation inaccuracy on the software side. The propulsive lifting velocity (Vprop), by definition, shares a significant overlap with the mean lifting velocity (Vmean), differing solely in the criterion utilized to define the end of a repetition (Vprop: acceleration below 1g, Vmean: velocity equaling zero). Hence, it is unexpected and unclear why Enode Pro exhibited poorer precision in Vprop in comparison to Vmean, particularly for the slow velocity spectrum, which was not the case for the resulting velocity parameters of the Apple Watch (Figure 3). This observation highlights the significance and impact of the embedded segmentation algorithm on the software side, given that the underlying raw acceleration data are identical for both velocity parameters. Consistent with the literature [11,12,17,18,19], we found all three devices to be valid for measuring Vmean, with the exception of slow repetitions measured by the Enode Pro device.

The peak lifting velocity of a repetition differs from the mean and propulsive velocities in that it is heavily reliant on the segmentation algorithm and the starting frame when differentiating the acceleration data. Even slight shifts in the segmentation spanning a few time frames may result in substantial errors. This challenge is further compounded when employing a real-time segmentation algorithm, as opposed to post-processing the acceleration data using offline segmentation. Additionally, peak velocity represents a singular snapshot of a time series, whereas mean velocity is calculated as the average across the entire concentric phase of the movement.

### 4.2. Connectivity and Segmentation

To achieve optimal compliance for various applications such as training interventions, prevention programs, and rehabilitation, it is essential that an athlete experiences as little frustration and exhaustion as possible in their use of technological devices. In the present work, connection issues that resulted in the omission of multiple repetitions were experienced using all devices. The progression of time revealed an increase in connectivity difficulties with the Apple Watch, leading to an increasing number of missed recordings when worn on the wrist. These accumulated connectivity issues were likely caused by the experimental protocol, which necessitated the execution of a calibration movement using the wrist-worn Apple Watch, which then prolonged the recording period. The actual source of the connectivity issue remains, however, unclear, and may have originated from either the Bluetooth connection between the Apple Watch and the iPhone or the network connection between the iPhone and the NodeJs server, respectively. However, the author is confident that the occurrence of such missed repetitions can be reduced with a more refined recording methodology executed by trained professionals.

Furthermore, it is noteworthy that the offline application of the present segmentation algorithm yielded only two (barbell) and three (wrist) inaccurately segmented repetitions, thereby corroborating the robustness of the applied algorithm and the results obtained. For the Enode Pro device, a total of 36 repetitions (accounting for 6.0% of the total) were missed due to connection issues. These results align with previous findings by Jukic et al. (free-weighted back squats, 184 repetitions, 4.6%), Feuerbach et al. (Smith machine back squats, 50 repetitions, 5%), and Fritschi et al. (different free-weighted exercises, 35 repetitions, 5.0%) [12,19,24]. Here, Jukic et al., suggested that Bluetooth-related problems may be the underlying cause of the missed repetitions [24].

Additionally, the presence of a considerable number of “ghost repetitions”, (i.e., caused by the re-racking of the barbell) indicates that the Enode Pro has implemented a highly sensitive segmentation algorithm for squat-specific exercise recognition, which may have resulted in inaccuracies, particularly during slow, unsteady and jittery repetitions. Setting a larger threshold for the minimal range of motion (ROM), which was possible in the Enode Pro app for individually created exercises (the exercise “barbell back squat” was chosen in this study), may have prevented these ghost repetitions. The version (1.1.5) of the Enode Pro app has addressed the issue of “ghost repetitions” in the beta version, and further studies should be sure to incorporate these changes. The algorithm developed for this study for the Apple Watches includes a correction for the device’s high sensitivity during segmentation, achieved by applying a minimal ROM threshold (0.3 m). Therefore, we hypothesize that by adjusting the minimal ROM, this low precision could be improved.

The extent of measurement errors encountered, particularly with the Enode Pro, was unexpected. Based on the validity outcomes, it is hypothesized that the issue may be attributed to software-related factors, given the challenges associated with the task of segmentation, especially in real time. Our app version 1.1.6 and firmware version 5.2.0 differ from those utilized in other studies [12,19,24]. Furthermore, given the potential software-related nature of the issue, it is crucial to report both the firmware and software versions of the devices utilized for validation purposes, to enable meaningful comparisons between publications.

### 4.3. Placement of VBT Device

Our study demonstrates that both the wrist-worn and barbell-mounted Apple Watch exhibit high precision for Vmean and Vprop parameters across the full velocity spectrum. While the precision is slightly inferior to that reported by Fritschi et al. [12], who analyzed the effect of the attachment point on the barbell, it still falls within an acceptable range (Table 3, SEE < 0.1 m/s, correlation coefficient (r) > 0.9). One explanation for the higher validity of the barbell-mounted Apple Watch compared to the wrist could be the potential accumulation of interference caused by the movement of the arm during the exercise, which is not present when using a rigid barbell. This is supported by the results of the Enode Pro, which are slightly worse compared to the barbell-mounted Apple Watch, and superior to the wrist-worn Apple Watch. However, the utilization of an Apple Watch worn on the wrist has the potential benefit of enhanced user-friendliness, time-efficiency, and cost-effectiveness in VBT. This is particularly relevant given that Thompson et al. [35] identified the significant drivers for coaches to purchase VBT technology as the combination of a good price point and functionality, emphasizing the crucial importance of user-friendliness. Nonetheless, to achieve higher levels of precision, as may be necessary in the case of coaching professional athletes or for research, we recommend attaching the IMU device directly to the barbell, using a secure fixation mechanism that minimizes motion relative to the barbell.

### 4.4. Limitations

In the current investigation, each of the three systems, namely the Apple Watch, the Enode Pro device and the Vicon system, were recording data autonomously, and the resulting velocity parameters were derived separately for each data modality. There was no synchronization established among these devices during data recording, which may have introduced some degree of variability into the results. The Enode Pro device, in adherence to standard usage protocols, was secured solely via its magnetic backside, which might have caused some inaccuracies due to device movement during squat performance testing. Due to practical constraints, the positioning of the Enode Pro device was not central, but rather offset from the midpoint of the barbell, contrary to the centrally located Apple Watch (see Figure 2). This divergent positioning might have introduced minor discrepancies in the collected measurement data, as suggested by previous research [12]. Yet, the comparison of the velocity parameters of the Enode Pro device was a secondary objective of the present work, with the primary focus being the assessment of the validity of the Apple Watch for measuring barbell kinematics.

## 5. Conclusions

The use of the IMU sensor in the Apple Watch as a tool for monitoring movement velocities during free-weighted back squats has shown promising results in terms of its validity. If these results are replicated by other studies, they could lead to the development of an affordable and user-friendly VBT device. However, it is important for practitioners to take into account the impact of the attachment site and the adopted segmentation algorithm on the precision of the resulting velocity parameters.

Our study confirmed previous research on the validity of the Enode Pro device, but also raised questions regarding repetitions at slow lifting velocities, which warrant further investigation. In terms of validity rankings across all 599 repetitions that were analyzed in this study, the barbell-mounted Apple Watch was found to be the most valid for assessing propulsive and peak lifting velocity, followed by the wrist-worn Apple Watch and the barbell-mounted Enode Pro device. For mean lifting velocities, the barbell-mounted Apple Watch led to the most accurate results compared to Vicon as the criterion, with slightly less accurate but similar results obtained for the barbell-mounted Enode Pro and wrist-worn Apple Watch. Furthermore, we emphasize the importance of disclosing the results of both software and hardware versions in scientific reports, as changes in validity may occur between versions.

## Figures and Tables

**Table 1:** Descriptive summary of recorded repetition data. Data pairs are displayed as the total numbers (n) over all three velocity spectra. A total of 599 repetitions were performed during data collection, of which 19 barbell-mounted and 49 wrist-worn Apple Watch repetitions were missed due to connection issues with the Nodejs server. A total of 70 repetitions were missed by the Enode Pro device due to connection issues, segmentation errors (leading to duplication or neglection of repetitions), or following the validity check (Vpeak > Vmean). The Enode Pro data of one participant were excluded from further analysis because of the large deviation in their squat technique.

Device	Total	Fast	Slow	Connection Issue	Segmentation Error	Validity Check	Ghost Reps	Participant Excluded	Handling Error
AW Barbell	578	290	288	19	2	-	-	-	-
AW Wrist	547	282	265	49	3	-	-	-	-
Enode Pro Barbell	496	258	238	36	17	17	25	1 (27 reps)	6

**Table 2:** VBT devices in comparison to Vicon as the criterion: calibration equation parameters with confidence limits (in brackets), standard error of estimate (SEE), and correlation coefficient (r). The slope, intercept and SEE were calculated using OLP regression, as outlined in the method section.

Accuracy					
Device	Parameter	Slope	Intercept	SEE (m/s, %)	Correlation Coefficient (r)
AW Barbell	Vmean	1.022	0.001	0.049	0.976
		[1.004, 1.041]	[−0.010, 0.012]	8.1%	[0.971, 0.979]
	Vpeak	1.112	0.023	0.092	0.959
		[1.087, 1.138]	[−0.006, 0.052]	7.2%	[0.952, 0.965]
	Vprop	1.045	−0.006	0.05	0.977
		[1.027, 1.064]	[−0.016, 0.004]	8.3%	[0.973, 0.981]
AW Wrist	Vmean	1.016	−0.008	0.064	0.959
		[0.992, 1.041]	[−0.023, 0.007]	10.4%	[0.952, 0.965]
	Vpeak	1.105	0.041	0.114	0.934
		[1.072, 1.139]	[0.003, 0.078]	8.8%	[0.922, 0.944]
	Vprop	1.029	−0.013	0.062	0.964
		[1.006, 1.052]	[−0.027, −0.0]	10.4%	[0.957, 0.969]
Enode Pro Barbell	Vmean	1.065	−0.048	0.059	0.966
		[1.041, 1.090]	[−0.063, −0.033]	9.6%	[0.959, 0.971]
	Vpeak	0.967	0.185	0.188	0.833
		[0.921, 1.016]	[0.131, 0.237]	14.8%	[0.804, 0.858]
	Vprop	0.990	0.038	0.113	0.882
		[0.949, 1.032]	[0.008, 0.055]	19.1%	[0.860, 0.900]

**Table 3:** Analysis of the effects of placement (wrist vs. barbell) of the Apple Watch. Calibration equation parameters with confidence limits (in brackets), standard error of estimate (SEE), and correlation coefficient (r). The slope, intercept and SEE were generated using OLP regression, as outlined in the method section.

Wrist-Worn vs. Barbell-Mounted Apple Watch		
Parameter	SEE (m/s, %)	Correlation Coefficient (r)
Vmean	0.063	0.958
	10.6%	[0.950, 0.964]
Vpeak	0.09	0.951
	7.9%	[0.942, 0.958]
Vprop	0.06	0.963
	10.4%	[0.957, 0.969]

## Data Availability

The resulting data set is available under the link for Supplementary Materials.

## Supplementary Materials

The following supporting information can be downloaded at: https://www.mdpi.com/article/10.3390/sports11070125/s1, Supplementary Material S1: Detailed description of motion capture data processing steps. Supplementary Material S2: Detailed description of acceleration data processing steps. Supplementary Material S3: Complete data set. Table S1: Detailed results (table) including velocity zones (1.25 s < t ≤ 1.25 s) of the barbell-mounted Apple Watch. Table S2: Detailed results (table) including velocity zones (1.25 s < t ≤ 1.25 s) of the wrist-worn Apple Watch. Table S3: Detailed results (table) including velocity zones (1.25 s < t ≤ 1.25 s) of barbell-mounted Enode Pro device.
