# Supplementary material for: Velocity-Based Strength Training: The Validity and Personal Monitoring of Barbell Velocity with the Apple Watch
Source: Sports (Basel). 2023 Jun 23;11(7):125. doi: 10.3390/sports11070125 (PMC10383699; doi:10.3390/sports11070125)
Supplement: Supplementary file 1 [file sports-11-00125-s001.zip › Supplementary S1.pdf]

sports

MDPI

Supplementary S1: Detailed description of motion capture data processing steps

Two reflective markers were placed on each end of the barbell. After labelling, gaps were filled with one of the filling functions built into Vicon Nexus. Thereafter, marker data was processed using a custom Python script. The main steps in this script were as follows:

1. The positions and trajectories of both barbell ends were identified as the mean of the two markers or (if fewer than two were visible) of two juxtaposed markers.
2. Then the two points were weighted to result in a virtual measurement point with 60cm distance from the left side of the barbell.
3. Afterwards, the acceleration signal was filtered with a 4th order Butterworth filter.
4. Followed by two differentiations, to determine the velocity and position data of the signal.
5. Next, the repetitions were segmented by an automated algorithm:
    1. Each repetition was found by the position minima (turning point) which was manually set.
    2. For each turning point the end of the repetition was determined by the following position maximum. A validity check in form of minimum range-of-motion (30cm) was applied.
    3. The start of the repetition was found by defining zero-velocity zones (+/- 0.06m/s) prior to the turning point. The last frame of the zero-velocity zones was defined as start. To exclude the sticking point from these potential zero-velocity zones the vertical displacement was also included, a minimal displacement difference of 30 cm to the turning point had to be existing. In order to eliminate the potential impact of the stretch-reflex (bounce) on the definition of Vmax, a minimum time threshold of 0.4 seconds was implemented.
6. For each repetition Vmean, Vpeak and Vprop were extracted and condensed into a csv file.
