# Supplementary material for: Velocity-Based Strength Training: The Validity and Personal Monitoring of Barbell Velocity with the Apple Watch
Source: Sports (Basel). 2023 Jun 23;11(7):125. doi: 10.3390/sports11070125 (PMC10383699; doi:10.3390/sports11070125)
Supplement: Supplementary file 1 [file sports-11-00125-s001.zip › Table S1.pdf]

Table S1 Velocity parameters of the barbell-mounted Apple Watch in comparison to Vicon as criterion: Calibration equation parameters with confidence limits (in bracket), Standard Error of Estimate (SEE) and Correlation Coefficient (r). Slope, Intercept and SEE were calculated using OLP regression as outlined in the method section.

Apple Watch Barbell

**Device**	**Velocity zone**	**Slope**	**Intercept**	**SEE (ms^{-1},%)**	**Pearson's r**
V mean	slow (t>=1.25s)	0.949 [0.905, 0.996]	0.03 [0.010, 0.048]	0.040 9.4%	0.911 [0.889, 0.929]
	fast (t<1.25s)	1.023 [0.984, 1.065]	0.002 [-0.029, 0.032]	0.055 7.0%	0.940 [0.925, 0.952]
	total	1.022 [1.004, 1.041]	0.001 [-0.010, 0.012]	0.049 8.1%	0.976 [0.971, 0.979]
V peak	slow (t>=1.25s)	1.309 [1.260, 1.359]	-0.140 [-0.186, -0.096]	0.077 7.2%	0.947 [0.933, 0.957]
	fast (t<1.25s)	1.117 [1.066, 1.169]	0.000 [-0.071, 0.067]	0.101 6.7%	0.917 [0.897, 0.934]
	total	1.112 [1.087, 1.138]	0.023 [-0.006, 0.052]	0.092 7.2%	0.959 [0.952, 0.965]
V prop	slow (t>=1.25s)	0.959 [0.912, 1.008]	0.027 [0.007, 0.045]	0.04 10%	0.903 [0.879, 0.922]
	fast (t<1.25s)	1.053 [1.015, 1.092]	-0.01 [-0.039, 0.018]	0.055 7.1%	0.949 [0.936, 0.959]
	total	1.045 [1.027, 1.064]	-0.006 [-0.016, 0.004]	0.05 8.3%	0.977 [0.973, 0.981]
