# Supplementary material for: Velocity-Based Strength Training: The Validity and Personal Monitoring of Barbell Velocity with the Apple Watch
Source: Sports (Basel). 2023 Jun 23;11(7):125. doi: 10.3390/sports11070125 (PMC10383699; doi:10.3390/sports11070125)
Supplement: Supplementary file 1 [file sports-11-00125-s001.zip › Table S2.pdf]

Table S2 Velocity parameters of the wrist-worn Apple Watch in comparison to Vicon as criterion: Calibration equation parameters with confidence limits (in bracket), Standard Error of Estimate (SEE) and Correlation Coefficient (r). Slope, Intercept a

Apple Watch Wrist

Device	Velocity zone	Slope	Intercept	SEE (ms^{-1},%)	Pearson's r
V mean	slow (t>=1.25s)	0.919 [0.858, 0.985]	0.027 [-0.002, 0.054]	0.057 13.3%	0.824 [0.781, 0.859]
	fast (t<1.25s)	0.964 [0.921, 1.009]	0.039 [0.004, 0.072]	0.063 8.1%	0.922 [0.902, 0.938]
	total	1.016 [0.992, 1.041]	-0.008 [-0.023, 0.007]	0.064 10.4%	0.959 [0.952, 0.965]
V peak	slow (t>=1.25s)	1.307 [1.234, 1.385]	-0.136 [-0.209, -0.068]	0.112 10.4%	0.879 [0.849, 0.904]
	fast (t<1.25s)	1.060 [1.005, 1.118]	0.090 [0.014, 0.163]	0.116 7.9%	0.891 [0.864, 0.913]
	total	1.105 [1.072, 1.139]	0.041 [0.003, 0.078]	0.114 8.8%	0.934 [0.922, 0.944]
V prop	slow (t>=1.25s)	0.916 [0.854, 0.982]	0.027 [-0.000, 0.053]	0.056 13.8%	0.818 [0.774, 0.855]
	fast (t<1.25s)	0.996 [0.956, 1.038]	0.017 [-0.015, 0.047]	0.061 7.9%	0.937 [0.921, 0.950]
	total	1.029 [1.006, 1.052]	-0.013 [-0.027, -0.0]	0.062 10.4%	0.964 [0.957, 0.969]
