# Supplementary material for: Velocity-Based Strength Training: The Validity and Personal Monitoring of Barbell Velocity with the Apple Watch
Source: Sports (Basel). 2023 Jun 23;11(7):125. doi: 10.3390/sports11070125 (PMC10383699; doi:10.3390/sports11070125)
Supplement: Supplementary file 1 [file sports-11-00125-s001.zip › Table S3.pdf]

Table S3 Velocity parameters of the barbell-mounted Enode pro device in comparison to Vicon as criterion: Calibration equation parameters with confidence limits (in bracket), Standard Error of Estimate (SEE) and Correlation Coefficient (r). Slope, Intercept a

Enode Pro Barbell

Device	Velocity zone	Slope	Intercept	SEE (ms-1,%)	Pearson's r
V mean	slow (t>=1.25s)	1.195 [1.086, 1.316]	-0.110 [-0.164, -0.061]	0.078 18.4%	0.660 [0.582, 0.726]
	fast (t<1.25s)	0.991 [0.965, 1.018]	0.013 [-0.008, 0.033]	0.036 4.6%	0.976 [0.969, 0.981]
	total	1.065 [1.041, 1.090]	-0.048 [-0.063, -0.033]	0.059 9.6%	0.966 [0.959, 0.971]
V peak	slow (t>=1.25s)	1.017 [0.930, 1.113]	0.123 [0.036, 0.202]	0.184 17.6%	0.710 [0.641, 0.768]
	fast (t<1.25s)	0.846 [0.778, 0.920]	0.362 [0.264, 0.451]	0.180 12.2%	0.731 [0.668, 0.783]
	total	0.967 [0.921, 1.016]	0.185 [0.131, 0.237]	0.188 14.8%	0.833 [0.804, 0.858]
V prop	slow (t>=1.25s)	1.001 [0.884, 1.134]	-0.009 [-0.064, 0.039]	0.114 28.2%	0.234 [0.110, 0.350]
	fast (t<1.25s)	0.756 [0.718, 0.796]	0.231 [0.203, 0.258]	0.076 9.9%	0.907 [0.883, 0.927]
	total	0.990 [0.949, 1.032]	0.032 [0.008, 0.055]	0.113 19.1%	0.882 [0.860, 0.900]
