# Supplementary material for: Velocity-Based Strength Training: The Validity and Personal Monitoring of Barbell Velocity with the Apple Watch
Source: Sports (Basel). 2023 Jun 23;11(7):125. doi: 10.3390/sports11070125 (PMC10383699; doi:10.3390/sports11070125)
Supplement: Supplementary file 1 [file sports-11-00125-s001.zip › Supplementary S2.pdf]

Supplementary S2: Detailed description of acceleration data processing steps

Regardless of the placement (wrist, barbell) the data from the Apple Watch was always handled identically.

The IMU data was collected via the Node.js application and stored accordingly. The raw data was then reduced to acceleration and orientation (acceleration + quaternions) entries and organized accordingly with a python script. Afterwards, the data got segmented as described below.

The main steps of the exercise segmentation in this script were as follows:

1. The vertical acceleration data was determined by a vector rotation of the device acceleration and device orientation (quaternions). These two parameters were generated by the Apple Watch.
2. The acceleration signal was filtered with a 4th order Butterworth filter, to remove jittering.
3. Double integration to get velocity and position data. Drift compensation was handled with subtraction of mean value.
4. Next, the repetitions were segmented by an automated algorithm:
    1. The identification of each repetition was based on the criterion of the maximum velocity, which was required to fall within the range of 0.3 to 2 m/s.
    2. Subsequently, a validity check was conducted to eliminate false maxima that were too close to one another to be considered a single repetition. 200 frames, which equals as 2 seconds was considered as too close. In such cases, the maxima with the higher velocity was deemed to be the true maximum.
    3. In this step, the end of repetition was determined. For this the positional maxima following the maxima in step 1 was looked for. As a validity check, the vertical positional difference between the maxima and the nearest position minimum (i.e., turning point) was required to be greater than 0.3m, which was defined as the minimal range of motion.
    4. To determine the turning point a window size of 8 seconds was defined and acceleration data was double integrated, backwards for 8 seconds to reduce drift. The positional minima was then obtained from this window.
    5. To identify the starting point, a 6-second window size was defined and the acceleration data was integrated backwards to obtain the velocity section. A threshold of 0.2m/s was initially defined for the starting point, and it was gradually reduced in 4 steps to 0.02 m/s. The starting point was determined as the first frame with a velocity less than 0 minus threshold. Therefore, if drift was too extreme, the starting point was less exact, but for most of the repetitions a threshold of 0.02 m/s was reached.
    6. In order to eliminate the potential impact of the stretch-reflex (bounce) on the definition of Vmax, a minimum time threshold of 0.4 seconds was implemented.
    7. Finally, a validity check was executed to verify that repetitions were not too close or overlapping.
5. For each repetition Vmean, Vpeak and Vprop were extracted and condensed into a csv file.
